# Nanoparticles Containing Curcumin Useful for Suppressing Macrophages *In Vivo* in Mice

**DOI:** 10.1371/journal.pone.0137207

**Published:** 2015-09-11

**Authors:** Chie Amano, Hideki Minematsu, Kazuyo Fujita, Shinki Iwashita, Masaki Adachi, Koichi Igarashi, Shuji Hinuma

**Affiliations:** 1 R&D Division, Katayama Chemical Industries, Co., Ltd., Minoh-City, Osaka, Japan; 2 Department of Food and Nutrition, Faculty of Human Life Science, Senri Kinran University, Suita-City, Osaka, Japan; Okayama University, JAPAN

## Abstract

To explore a novel method using liposomes to suppress macrophages, we screened food constituents through cell culture assays. Curcumin was one of the strongest compounds exhibiting suppressive effects on macrophages. We subsequently tried various methods to prepare liposomal curcumin, and eventually succeeded in preparing liposomes with sufficient amounts of curcumin to suppress macrophages by incorporating a complex of curcumin and bovine serum albumin. The diameter of the resultant nanoparticles, the liposomes containing curcumin, ranged from 60 to 100 nm. Flow cytometric analyses revealed that after intraperitoneal administration of the liposomes containing curcumin into mice, these were incorporated mainly by macrophages positive for F4/80, CD36, and CD11b antigens. Peritoneal cells prepared from mice injected in vivo with the liposomes containing curcumin apparently decreased interleukin-6-producing activities. Major changes in body weight and survival rates in the mice were not observed after administrating the liposomes containing curcumin. These results indicate that the liposomes containing curcumin are safe and useful for the selective suppression of macrophages in vivo in mice.

## Introduction

Macrophages have a variety of functions as follows. (1) Primary self-defense through phagocytosis of pathogens and dead cells [1]. (2) Secondary immune reactions through antigen presentation by displaying processed antigens together with major histocompatibility complex molecules [2]. (3) Production of various cytokines including interleukin-1 (IL-1), interleukin-6 (IL-6), tumor necrosis factor (TNF), and others [3]. In addition, recent studies indicate that macrophages play important roles in the progress of some diseases including diabetes [4], cancer [5], and arteriosclerosis [6]. To clarify the in vivo roles of macrophages in animal models, one of the most effective approaches is to suppress macrophages in vivo in a specific manner. For this purpose, liposomes containing clodronate (clodronate/liposome) have been used as a conventional method [4, 5]. Clodronate is a synthetic bisphosphonate originally developed for an anti-osteoporotic drug. This compound is expected to selectively suppress osteoclasts, a kind of macrophage. Liposomes are artificial vesicles with sizes at nanometer or micrometer levels that mimic the lipid bilayer of the cell membrane. Because of excellent biocompatibility and biodegradability, liposomes are presumed to be potential candidates as carriers of drug delivery systems (DDS). As to the fate of liposomes injected in vivo, the accumulated evidence indicates that the predominant uptake of liposomes takes place in the reticuloendotherial system (RES), that is, in principal macrophages [7, 8]. Macrophages thus mainly capture clodronate/liposome after in vivo injection. Indeed, clodronate/liposome could suppress macrophages efficiently in vivo in animal models [4, 5, 9]. However, clodronate/liposome has a problem concerning toxicity, for example, intraperitoneal (i.p.) administration of clodronate/liposome at a dose necessary for suppressing macrophages caused rapid death in mice (unpublished data).

To overcome such a problem with clodronate/liposome, we searched for candidate compounds that can be substituted for clodronate. We employed food constituents as target compounds, because it is conceivable that they are relatively safe and tolerable based on their history of human consumption. In the course of this study, we confirmed that curcumin (diferuloylmethane) could be a potential compound for suppressing macrophages. We thus prepared liposomes containing curcumin. Curcumin is a major constituent of the spice turmeric (*Curcuma longa*), yellow colored dye, and has various biological activities [10]. Although some approaches to prepare liposomal curcumin has been reported [11–13], its exact physicochemical properties and in vivo suppressing activities against macrophages are still unclear. In this paper, we will report a novel method using a curcumin and bovine serum albumin (BSA) complex (curcumin/BSA) to prepare liposomes containing a high amount of curcumin and their physicochemical and bioactive properties. We will demonstrate here that nanoparticles containing curcumin/BSA, which we have established a preparation method, is useful for suppressing macrophages in vivo in mice.

## Materials and Methods

### Animal care and use

Experiments using mice were carried out in accordance with the guidelines for Proper Conduct of Animal Experiments, Science Council of Japan (Tokyo, Japan). A committee on animal experiments in Katayama Chemical Industries, Co., Ltd. granted permission for the animal use its experimental procedure in this study.

Balb/c mice were obtained from Japan SLC, Japan. Balb/c mice (6-week old, female) used for experiments were housed in a 12 h light/dark cycle in a temperature-controlled environment. They were fed normal diets (Oriental Yeast, Japan) ad libitum.

In experiments to examine survival rate and body weight changes in mice after the administration of liposomes, we determined the humane endpoint by 8 days if the mice appeared to be dying due to severe weight loss (approximately more than 20%). We monitored the conditions of the mice every day during the experiments for 8 days. However, these mice had no severe body weight loss and appeared good conditions during the period monitored. At the experimental endpoint (8 days after the administration of liposomes), mice were humanely euthanized to minimize suffering through cervical dislocation by an expert. In experiments to use mouse peritoneal cells, they were prepared from mice anesthetized with isoflurane. After finishing the preparation of peritoneal cells, these mice were immediately euthanized through cervical dislocation with expertise.

### Cell proliferation assays

We used THP-1 (a human monocytic leukemia cell line), L929 (a mouse fibroblast cell line), RAW264.7 (a mouse macrophage cell line), and mouse bone marrow-derived macrophages [14] cultured in a flask or plate at 37°C, under a humidified atmosphere of 5% CO_2_ in air. THP-1 and L929 were obtained from Japan Health Science Foundation. RAW264.7 was purchased from DS Pharma Biomedical Co., Ltd. These cells were cultured in RPMI1640 + 10% FCS consisting of RPMI1640 [+] L-glutamine (Gibco) supplemented with antibiotics (penicillin and streptomycin; Gibco) and 10% fetal calf serum (FCS; Biowest). In cell proliferation assays, they were cultured in flat-bottomed 96-well plates (Corning) in 100 μl/well at a concentration of 1 x 10^5^/ml with or without test samples. In the case of mouse peritoneal cells, we employed 1 x 10^6^/ml as a cell concentration. After finishing the culture, we added 10 μl of a coloring reagent, WST-8 (Kishida Chemical Co.), which converts to water-soluble colored formazan by the action of a mitochondrial enzyme. Cells were additionally incubated with WST-8 for 3 h and the coloring reaction was stopped by adding 10 μl of 0.1% sodium dodecyl sulfate (SDS) solution. The absorbance of each well was then measured at 450 nm with a Multiskan Ascent plate reader (Labsystems). In principle, we performed cell proliferation assays in duplicate. Half-maximal (50%) inhibitory concentration (IC_50_) of each test sample was calculated according to the reported formula [15].

### Test compounds

We examined the inhibitory activities of food constituents in cell proliferation assays. Test compounds used in the assays were as follows: curcumin (Wako or Sigma-Aldrich), zerumbone (Wako), genistein (Sigma-Aldrich), chalcone (Wako), corosolic acid (Wako), carnosol (Wako), ursolic acid (Wako), epigallocatechin (Sigma-Aldrich), isoflavone (Wako), apigenin (Wako), chrysin (Wako), flavanone (Wako), resveratrol (Wako), flavone (Wako), quercetin (Wako), daidzein (Wako), and clodronate (Sigma-Aldrich). These compounds were firstly dissolved with dimethyl sulfoxide (DMSO; Wako) and then appropriately diluted with RPMI1640 + 10% FCS to apply the assays. The final concentration of DMSO was adjusted not to exceed 1% at most in cell cultures.

### Preparation of liposomes containing a complex of curcumin and albumin

BSA (Sigma-Aldrich) or human serum albumin (HSA; Sigma-Aldrich) was dissolved in 10 mM phosphate buffer (pH 8.0) at 40 mg/ml. Curcumin was dissolved in DMSO at 10 mg/ml. Both albumin and curcumin solutions were mixed at 50/50 by weight to prepare curcumin absorbed by HSA (curcumin/HSA) or BSA (curcumin/BSA), and then the mixture was filtrated with the phosphate buffer by an ultrafiltration filter (Ultracel 10 kDa; Millipore) to remove DMSO. Precipitate generated during the ultrafiltration was removed by passing the mixtures through a 0.22 μm filter (Millex-GV filter unit, PVDF; Millipore).

To prepare liposomes, we tried various techniques including freeze-drying [16, 17], remote loading [18, 19], Bangham [20], and improved cholate dialysis [21, 22] methods. Because the improved cholate method was the most efficient in producing nanoparticles containing a sufficient amount of curcumin, we employed mainly this method for liposomal formation of curcumin in this study. We dissolved 1, 2-dipalmitoyl-*sn*-glycero-3-phosphocholine (DPPC; NOF Co.), 1, 2-dipalmitoyl-*sn*-glycero-3-phospho-L-serine sodium salt (DPPS; Sigma), and cholesterol (NOF Co.) at the molar ratio of 5:1:4 (18 mg in total) and sodium cholate (18 mg) in 3 ml of methanol-chloroform (volume ratio 1:1). The solution was evaporated in an eggplant shaped flask, and then vacuum dried for 30 min to form a lipid film. The phosphate buffer (1 ml) was added to the film in the flask, and it was rotated at 37°C for 1 h. The flask was subsequently treated with a sonication bath (VS-100Ⅲ; AS ONE) for 1 h, and then 2 ml of curcumin/HSA or curcumin/BSA solution (1.6 mg/ml of curcumin) was admixed to the solution. Sodium cholate in the solution was subsequently removed in the phosphate buffer by filtration with an ultrafiltration filter (Ultracel 10 kDa; Millipore). After liposomal formation, free curcumin/HSA or curcumin/BSA was removed by ultrafiltration in a 4-(2-hydroxyethyl)-1-piperazine ethane sulfonic acid (HEPES; DOJINDO) buffer (pH 7.2) containing 0.9% NaCl (SAJ) with a filter (Biomax 300 kDa; Millipore). In initial experiments, we used curcumin/HSA to examine liposomal formation of curcumin. However, because there was no significant difference between curcumin/HSA and curcumin/BSA in the liposomal formation, we mainly used curcumin/BSA to prepare liposomal curcumin in this study. If necessary, the curcumin concentration of a curcumin/liposome suspension was properly adjusted according to each experiment’s purpose.

The particle size and zeta-potential of liposomes were measured through dynamic light scattering and electrophoretic light scattering respectively with Zetasizer nano ZSP (Malvern).

### Flow cytometric analyses

Liposomes were injected twice i.p. into Balb/c mouse 48 h and 72 h before harvesting peritoneal cells. Two hundred microliters of curcumin/liposome (1.8 mg/ml as a curcumin concentration) were injected per mouse, and control liposomes without curcumin were adjusted to the same lipid amount as curcumin/liposome. Peritoneal cells were pooled from three mice in each group 48 h after the last injection. Cell staining and flow cytometric analyses were performed with Guava easyaCyte (Merck) according to the method as described previously [23]. Peritoneal cells (1 × 10^7^/ml) were stained with Alexa Fluor 488 anti-mouse CD90.2 (Thy-1.2) Antibody(BioLegend)at 2.5 μg/ml, fluorescein isothiocyanate (FITC) rat anti-mouse F4/80(AbD serotec) at 10 μg/ml, Alexa Fluor 488 anti-mouse CD36 Antibody (BioLegend) at 2.5μg/ml, and PerCP/Cy5.5 anti-mouse/human CD11b (BioLegend) at 2.5 μg/ ml, respectively.

### IL-6 production assay

In in vitro assays, peritoneal cells were obtained from mice 3 days after i.p. administration of 5% (w/v) thioglycollate (Fluka) in 5ml of distilled water. These cells pooled in each group (three mice) were washed twice with a phosphate-buffered saline by centrifugation, and then suspended in RPMI1640 + 10% FCS at 1×10^6^/ml. The cell suspension (1 ml/well) was cultured in the presence of curcumin/liposome or control liposomes in a 12-well microplate (Corning) for 24 h under humidified 5% CO_2_ in air. IL-6 production from the cultured cells was examined in the presence or absence of lipopolysaccharide (LPS; Chondrex) at 0.5 μg/ml. After 24 h incubation, we harvested culture supernatants from the microplates, and then centrifuged these at 2,000 r.p.m. for 10 min to remove debris. The amounts of IL-6 in the culture supernatants were determined in triplicate with a mouse IL-6 ELISA kit (Tepnel Life Sciences).

To assess the effect of in vivo administering curcumin/liposome (2.9 mg/ml as curcumin concentration in 200 μl) on IL-6 production from peritoneal cells, we injected thioglycollate into mice in each group 3 days before harvesting peritoneal cells. Curcumin/liposome (three mice) or control liposomes (two mice) were injected 4 and 24 h before the cell harvest. We pooled peritoneal cells obtained from mice injected with liposomes, and then examined their IL-6-producing ability through in vitro culture assay under the same conditions as described above.

### Examination of body weight changes in mice

Curcumin/liposome was injected once i.p. or intravenously (i.v.) into Balb/c mice at two doses (1.5 and 2.9 mg/ml as curcumin concentrations in 200 μl), respectively. Untreated mice were used as a control. Five mice were used in each group in these experiments. We measured their body weight for 8 days.

## Results

### Screening of macrophage-suppressive compounds in food constituents

To develop a novel method using liposomal technology to suppress macrophages, we searched for candidate compounds to contain in liposomes through screening food constituents by cell proliferation assays. We screened multiple compounds in cell proliferation assays using THP-1, mouse bone marrow-derived macrophages, and L929 cells. As a result, we found that some compounds showed strong proliferation-inhibitory activities against these cells. Representative compounds with apparent inhibitory activities are listed in Table 1.

**Table 1:** Representative Compounds Showing Inhibitory Activities in Cell Proliferation Assays.

Test sample^a^	THP-1		Mouse bone marrow-derived macrophages		L929	
	IC_50_ ^b^ (μM)	n^c^	IC_50_ ^b^ (μM)	n^c^	IC_50_ ^b^ (μM)	n^c^
Curcumin	13.0 ± 3.2	3	22.4 ± 3.6	2	22.8 ± 10.8	2
Zerumbone	16.0 ± 0.5	2	32.1	1	30.4 ± 0.1	2
Genistein	16.0 ± 6.1	12	29.0 ± 21.5	4	28.2 ± 7.5	9
Chalcone	17.4 ± 1.8	2	103.6	1	69.5 ± 1.2	2
Corosolic acid	18.2 ± 0.2	2	19.6 ± 0.6	2	17.7 ± 0.2	2
Carnosol	20.5 ± 2.7	2	63.8 ± 5.5	2	50.1 ± 2.6	2
Ursolic acid	24.6 ± 3.4	2	16.3 ± 4.5	2	30.7 ± 0.8	2
Epigallocatechin	37.0 ± 29.6	3	157.4 ± 19.9	2	40.1 ± 3.7	2
Isoflavone	37.6 ± 11.6	2	105.9 ± 7.8	2	65.5 ± 3.6	2
Apigenin	37.9 ± 3.9	2	70.0 ± 19.8	2	46.4 ± 3.3	2
Chrysine	46.8 ± 2.8	2	58.2	1	76.3 ± 11.0	2
Flavanone	55.1 ± 10.9	2	225.0 ± 66.1	2	211.0 ± 29.6	2
Resveratrol	64.6 ± 8.5	3	77.2 ± 13.0	3	84.8 ± 29.1	3
Flavone	86.9 ± 4.1	2	180.5	1	34.5 ± 3.4	2
Quercetin	125.4 ± 24.1	2	126.2 ± 83.4	2	116.6 ± 32.5	2
Daidzein	>400	2	65.1 ± 33.5	2	219.6 ± 57.9	3
Clodronate	>400	8	25.7 ± 15.6	3	335.6 ± 54.9	7

^a^Test samples were dissolved in DMSO and then added to cell culture. Final concentration of DMSO was adjusted less than 1% in the culture. Under this condition, DMSO did not show inhibitory activities.

^b^Dose-response curves were obtained by two-fold serial dilution of test samples starting from 400 mM at most. In each experiment, each assay was done in duplicate. Data represents means ± range (n = 2) or standard deviation (n > 2) in repeated experiments.

^c^n represents the number of experiments done to obtain these data.

Among the compounds tested, curcumin, zerumbone, genistein, chalcone, corosolic acid, carnosol, and ursolic acid exhibited very strong inhibitory activities against the THP-1 cell proliferation at IC_50_ values less than 30 μM. Clodronate showed weaker activity on THP-1 and L929 cells, but comparable strong activity to mouse bone-marrow derived macrophages. In this screening, we preferentially used THP-1 to compare the activities in compounds, because THP-1 is a cell line originally established from human monocytic leukemia, and the results of the assay using the cell line are easily reproduced. In this screening, we also used mouse bone marrow-derived macrophages. However, this assay was difficult to obtain consistent results because each assay required new test cells to be prepared from fresh mouse bone marrow. We therefore decided to choose candidate compounds preferentially from the assay using THP-1. Curcumin was one of the strongest suppressive compounds against THP-1, mouse bone marrow-derived macrophages, and L929.

As curcumin appeared to have enough suppressive activity to at least both THP-1 and mouse bone marrow-derived macrophages when compared to clodronate, we chose this compound for liposomal formation. Although curcumin also showed strong inhibitory activity against the fibroblastic cell line (L929), we expected that liposomal curcumin would be trapped selectively by RES macrophages.

### Preparation of curcumin/liposome

As shown in S1 Table, we examined various techniques from (1) to (7) for incorporating curcumin solely into liposomes. However, we were unable to prepare liposomes that contained a sufficient amount of curcumin to suppress macrophages by these methods. We thus subsequently tried to make a complex of curcumin and albumin at the first step, and then prepare liposomes containing this complex at the second step as shown in (8) and (9). We examined which ratio between HSA and curcumin was the most efficient to form curcumin/HSA. We tested 10, 20, and 40 mg of curcumin dissolved in DMSO (1ml) to absorb 10 mg of HSA dissolved in a buffer (1 ml). Curcumin absorbed by HSA was estimated to be 38.3, 23.2, and 21.6 μg curcumin at 1mg HSA, respectively. Because the amount of absorbed curcumin was the highest by curcumin and HSA at the ratio of 1:1, we decided to use this ratio to prepare a curcumin and albumin complex. By employing this curcumin absorbed by albumin, we succeeded in preparing liposomes that contained enough amounts of curcumin to suppress macrophages. When curcumin/HSA or curcumin/BSA was used to prepare high content of curcumin in liposomes (curcumin/liposome), improved cholate dialysis method (0.512 of curcumin and lipid ratio) was more efficient than Bangham method (0.147 of curcumin and lipid ratio) as shown in S1 Table. We therefore decided to use improved cholate dialysis method for preparing liposomes with curcumin/HSA or curcumin/BSA. In five repeated experiments to prepare curcumin/liposome using curcumin/BSA, curcumin concentration of this suspension was 1.25 ± 0.44 (mean ± standard deviation) mg/ml. When curcumin/HSA and curcumin/BSA were compared, they showed almost the same results in the liposomal formation, so we employed curcumin/BSA to prepare curcumin/liposome in the following experiments.

**Fig 1:**
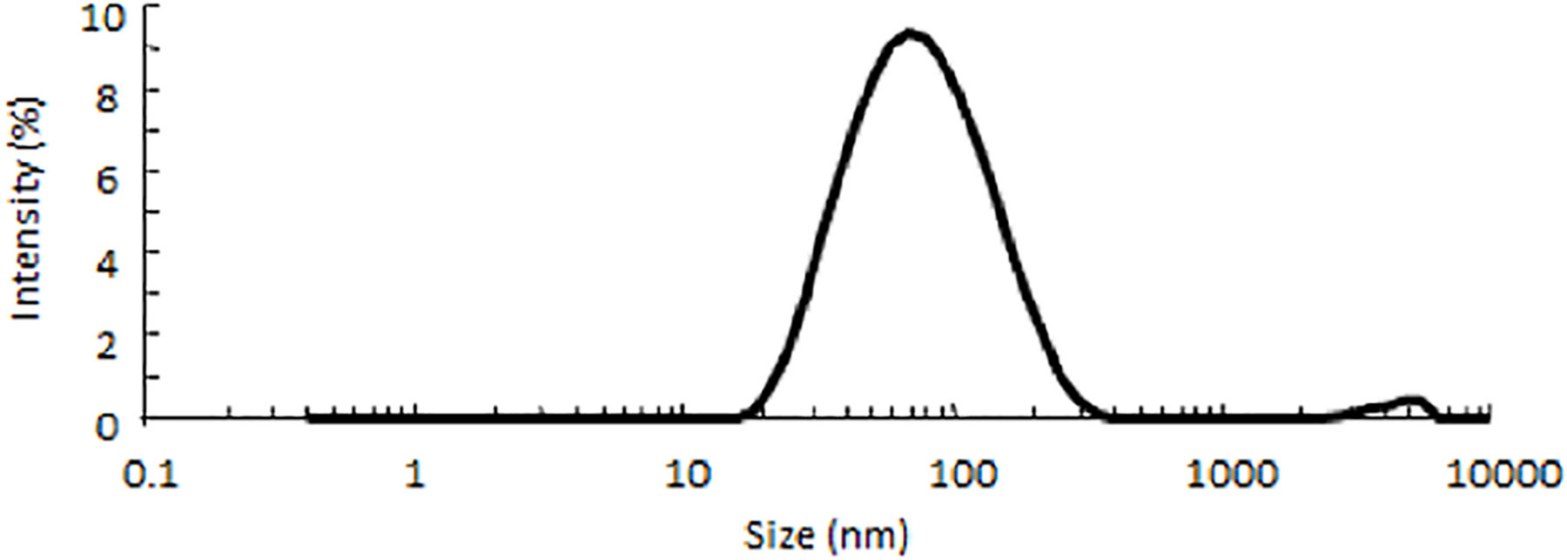
Size Distribution of Curcumin/Liposome. Representative data on the size of curcumin/liposome are shown here. The vertical axis shows the relative intensity of light scattering and the horizontal axis shows the size of liposomes on log scale.

We examined the particle size and zeta-potential of curcumin/liposome by dynamic light scattering and electrophoretic light scattering, respectively. As shown in Fig 1, its size ranged from 20 to 300 nm, and the main peak was detected at between 60 to 100 nm. The mean value of z-potential was 71.4 ± 9.0 (mean ± standard deviation) in four independent experiments. In this distribution, polydispersity index was 0.28 ± 0.02. Its zeta potential was -38.2 ± 4.3 mV, indicating that curcumin/liposome carried a negative charge.

### Effect of curcumin/liposome on proliferation of macrophage cell lines

**Fig 2:**
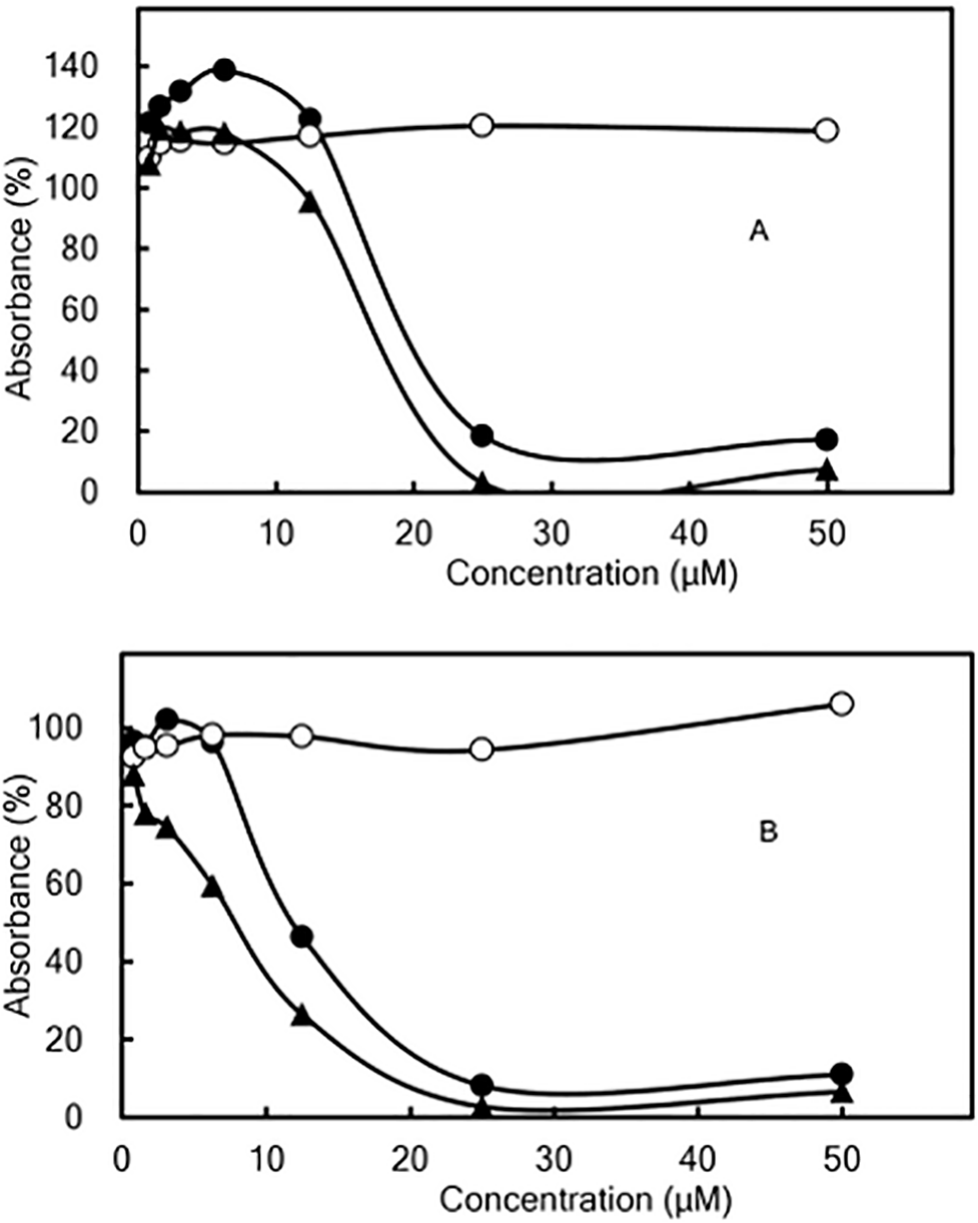
Dose-Response Curves of Curcumin/Liposome in Inhibition of Cell Proliferation. THP-1 (panel A) or RAW264.7 (panel B) cells were cultured for 3 days in the presence of curcumin/liposome (●), curcumin (▲), and control liposomes (○), respectively. After culturing, they were incubated with WST-8 for 3 h to assess viable cells in the culture. Resultant coloring reaction by WST-8 was measured with a plate-reader at the absorbance of 450 nm. In the vertical axis, the absorbance of only the medium was defined as 0%. Symbols in the Figure represent the percentage of absorbance with curucmin or liposomes to that without the test samples. Data represent mean values ± range (bars) in duplicate assays.

We examined whether curcumin/liposome retained suppressive activity against the proliferation of macrophage cell lines after liposomal formation. Curcumin/liposome showed dose-dependent inhibition of cell proliferation of THP-1 (Fig 2A) and RAW264.7 (Fig 2B) in a similar manner to curcumin, indicating that curcumin’s inhibitory activity did not drastically diminish through the process of nanoparticle formation.

### Cell surface markers of peritoneal cells incorporating curcumin/liposome

It has been reported that liposomes injected in vivo in mice were mainly incorporated by RES [7, 8]. To confirm whether macrophages in peritoneal cells actually incorporate curcumin/liposome, we analyzed cell surface markers on peritoneal cells by flow cytometry after i.p. administration of curcumin/liposome. Curcumin has a fluorescent property (emission wavelengths ranging from 470 to 700 nm by excitation wavelengths of less than 520 nm [24]). Fluorescence of curcumin is thus detectable in both green (525 ± 30 nm) and red (680 ± 30 nm) under these flow cytometric conditions.

**Fig 3:**
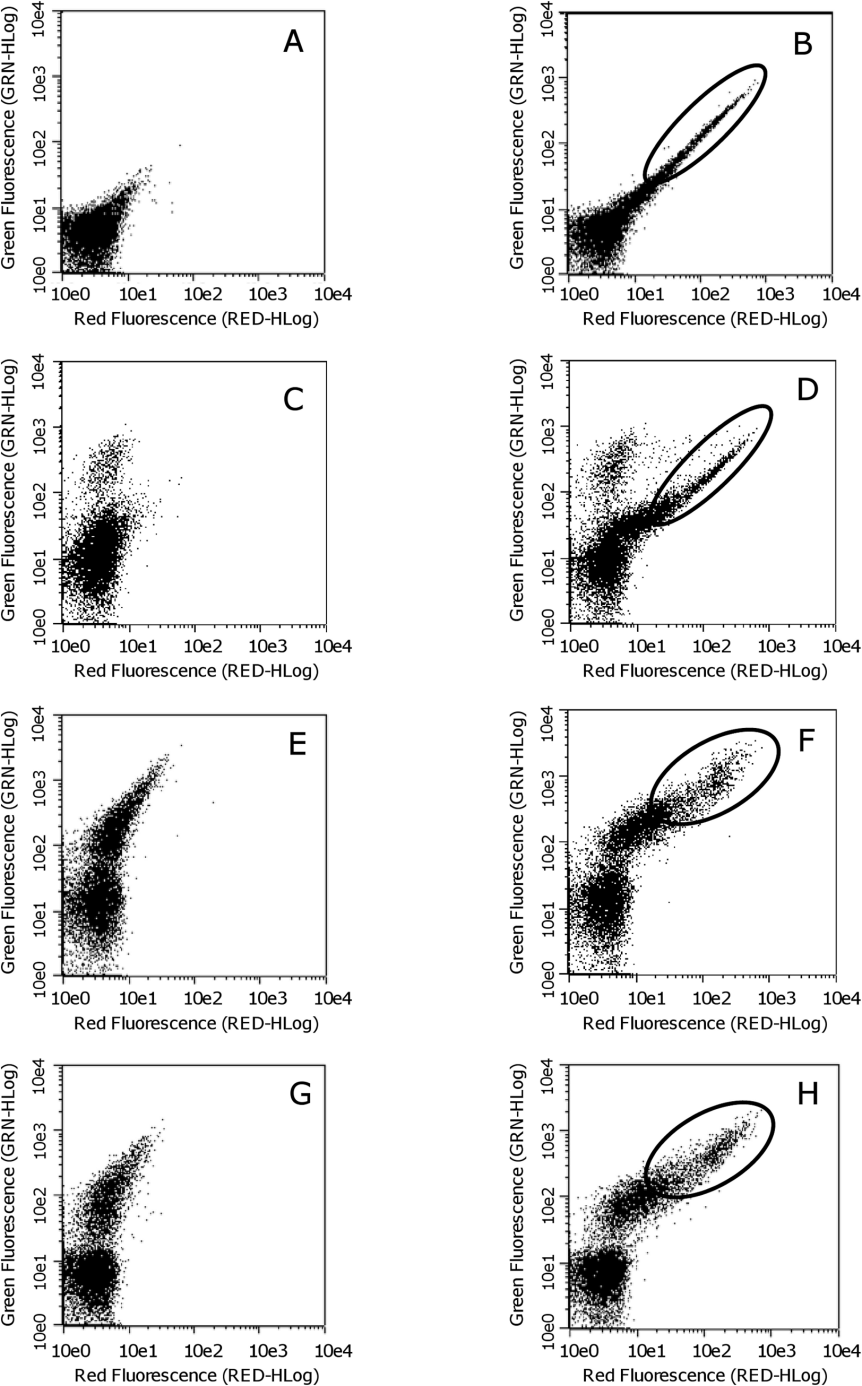
Flow Cytometric Analysis for Mouse Peritoneal Cells after Administration of Curcumin/Liposome. Panel A, C, E, and G show results obtained from mice injected twice with control liposomes (200 μl) 48 and 72 h before analysis. Panel B, D, F, and H show those from mice injected with curcumin/liposome (200 μl, 1.8 mg/ml of curcumin concentration) in the same manner. In panel A and B, peritoneal cells were not stained; in panel C and D, were stained with Alexa Fluor 488 anti-CD90.2 (Thy1.2) antibody; in panel E and F, were stained with FITC anti-F4/80 antibody; in panel G and H, were stained with Alexa Fluor488 anti-CD36 antibody. Dots plotted in the Figures show fluorescence intensity. The vertical axis and the horizontal axis represent green red fluorescence intensity, respectively. GRN-HLog or RED-HLog means the highest fluorescence intensity on log scale. Excitation was performed with a blue laser at 488 nm. Green fluorescence was detected with a 525 ± 30 nm band pass filter and red fluorescence with a 680 ± 30 nm band pass filter. It is notable that dots in the circled areas of panel F and H apparently show higher green fluorescence compared to panel B and D.

Fig 3A and 3B represent fluorescence of unstained peritoneal cells obtained from mice injected with control liposomes and curcumin/liposome, respectively. Double-positive cells with green and red fluorescence apparently increased in Fig 3B (indicated as circled area) than in Fig 3A, suggesting that some part of peritoneal cells apparently incorporated curcumin/liposome. We subsequently examined which type of cells incorporated curcumin/liposome, by means of cell surface antigens [CD90.2 (Thy 1.2), F4/80, CD36, and CD11b]. Thy1 is a representative T cell maker [23]. On the other hand, F4/80, CD36, and CD11b are known as macrophage cell surface makers [25, 26]. By staining with Alexa Fluor 488 anti-Thy1 antibody, a minor population of Thy1-positive cells were detected as the increase of green fluorescence in the peritoneal cells of mice injected with control liposomes (Fig 3C). In the peritoneal cells of mice injected with curcumin/liposome, the population of double-positive cells with green and red fluorescence seen in the circle of Fig 3D were detected in almost the same position in the circled area in Fig 3B even after the staining with Thy1, suggesting that the cells incorporating curcumin/liposome were negative for Thy1.

On the other hand, as shown in Fig 3E, when the cell staining was done with FITC-labeled anti-F4/80 antibody, F4/80-positive cells were found to exist mainly in the peritoneal cells of mice injected with control liposomes as indicated by the increase of green fluorescence. This result is consistent with the fact that macrophages are rich in the peritoneal fluid [27]. In addition, in peritoneal cells of mice injected with curcumin/liposome, curcumin fluorescence was detected in the circle illustrated in Fig 3F. When compared with the circled area of Fig 3B, dots in the circled area in Fig 3F evidently increased their green fluorescence, suggesting that F4/80-positive cells mainly incorporated curcumin/liposome. Similar results were obtained by using Alexa 488-labeled anti-CD36 antibody. As shown in Fig 3G, CD36-positive cells existed substantially in the peritoneal cells harvested from mice injected with control liposomes. In the peritoneal cells of mice injected with curcumin/liposome, curcumin fluorescence was detected in the circled area of Fig 3H. This result suggested that CD36-positive cells mainly captured curcumin/liposome. When we stained the peritoneal cells with PerCP/Cy5.5-labeled anti-CD11b antibody, similar results were obtained (data not shown). These results indicated that F4/80-, CD36-, CD11b-positive cells (i.e., macrophages) dominantly incorporated curcumin/liposome after its in vivo administration.

### Effect of curcumin/liposome on IL-6 production from peritoneal cells

**Fig 4:**
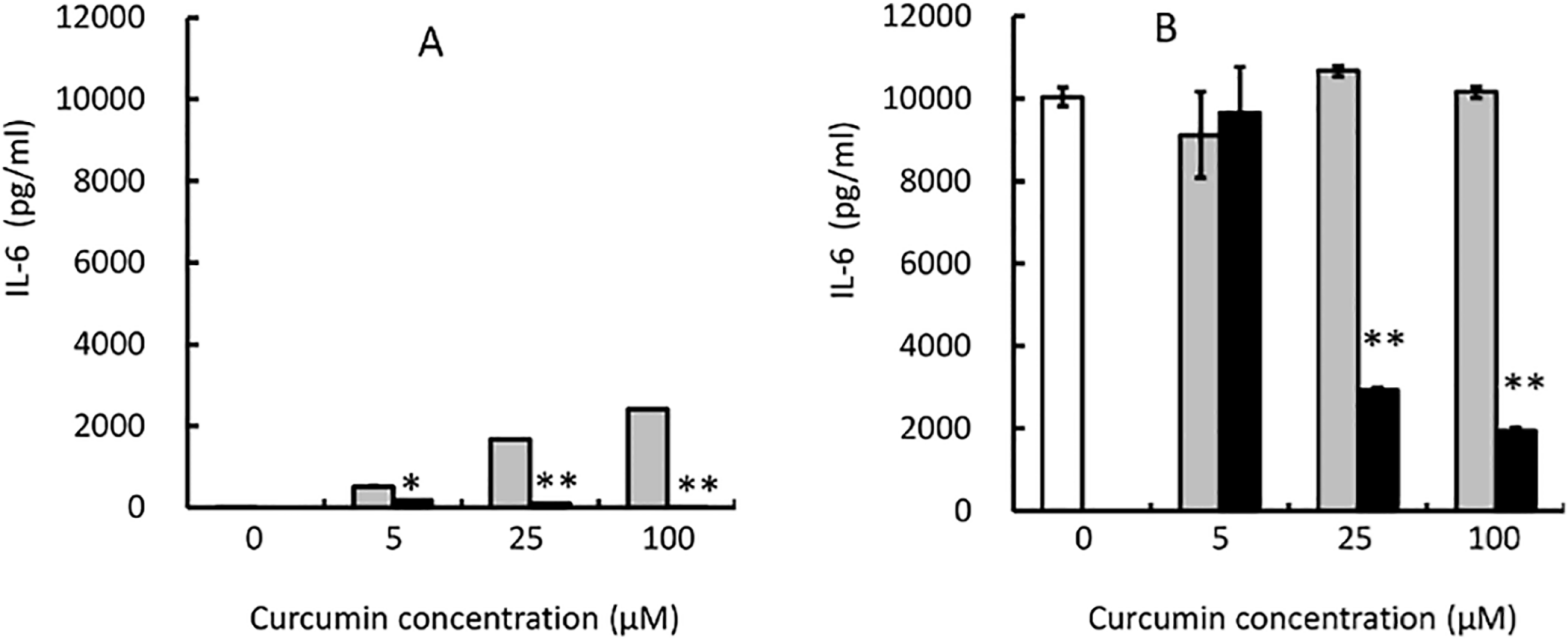
Effect of Curcumin/Liposome on IL-6 Production from Mouse Peritoneal Cells in In Vitro Culture. Peritoneal cells harvested from Balb/c mice injected with thioglycollate 3 days before. Peritoneal cells were cultured for 24 h in the absence (panel A) or presence (panel B) of LPS at 0.5 μg/ml. Blank columns represent cultures absent of both curcumin/liposome and control liposome. Curcumin/liposome (filled bars) was added to the culture so that curcumin concentrations became as indicated in the Figures. Control liposomes (gray bars) were added to culture in the same manner as curcumin/liposome, and its lipid concentration was consistent with that of curcumin/liposome. Data represent mean values ± standard deviation (bars) in triplicate assays. The statistical analysis was carried out by the standard Student’s t-test. * and **indicate that P<0.05 and P<0.01 compared to control liposomes, respectively.

We examined whether curcumin/liposome could suppress IL-6 production from peritoneal cells because macrophages are one of the main producers of IL-6 in the body [28]. We collected peritoneal cells from mice injected with thioglycollate. In culture for 24 h without a stimulator, LPS, peritoneal cells produced little IL-6 (Fig 4A). The addition of empty liposomes seemed to induce slightly IL-6 production from peritoneal cells. However, curcumin/liposome suppressed IL-6 production when compared to empty liposomes, suggesting that curcumin has some function to suppress IL-6 production evoked by liposome. Under culture conditions in the presence of LPS, peritoneal cells produced high amounts of IL-6 (Fig 4B). Curcumin/liposome evidently suppressed IL-6 production of mouse peritoneal cells in a dose-dependent manner (Fig 4B).

**Fig 5:**
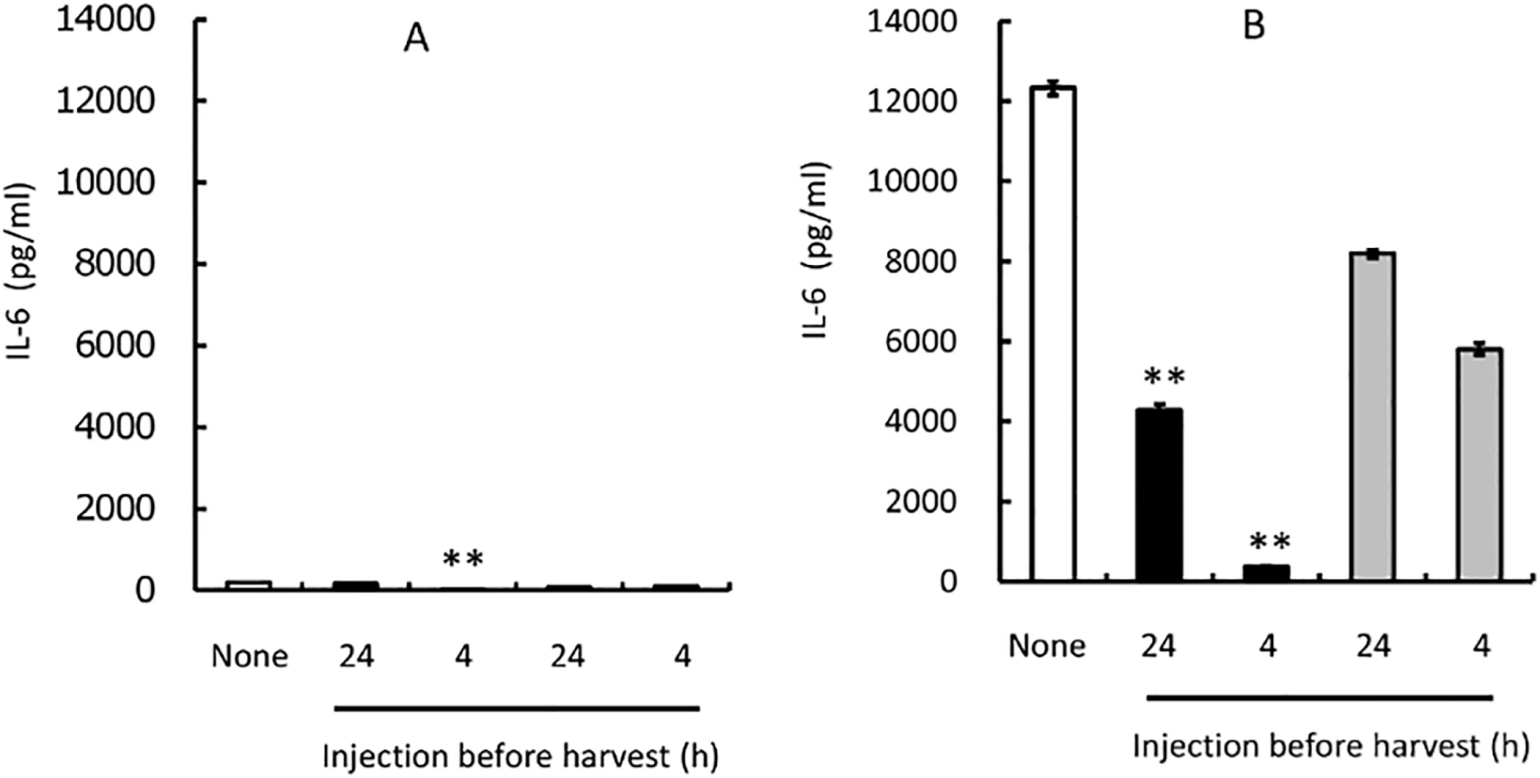
Effect of In Vivo Administration of Curcumin/Liposome on IL-6 Production from Mouse Peritoneal Cells. Balb/c mice were injected with thioglycollate 3 days before harvesting peritoneal cells. Curcumin/liposome (filled bars) or control liposomes (gray bars) were injected into the mice 4 or 24 h before harvesting the cells. The harvested cells were cultured for 24 h in the absence (panel A) or presence (panel B) of LPS as described in Fig 4. Blank columns represent IL-6 production in the culture of the peritoneal cells from mice without receiving the injection of liposomes. IL-6 in the culture supernatants were quantified in triplicate assays. Data represent mean values ± standard deviation (bars) in triplicate assays. **indicate that P<0.01 compared to mice injected with control liposomes.

To determine whether curcumin/liposome could suppress macrophage function in vivo in mice, we administered curcumin/liposome (200 μl, 2.9 mg/ml as curcumin concentration) i.p. into mice, and then determined the ability of IL-6 production in peritoneal cells. Mice used for this assay were treated with thioglycollate to induce macrophages into the peritoneal cavity before injecting liposomes. Curcumin/liposome or control liposomes were injected into each mouse 4 and 24 h before harvesting peritoneal cells, respectively. As shown in Fig 5A, peritoneal cells produced few amounts of IL-6 in culture in the absence of LPS. IL-6 productions in the culture were apparently enhanced by the addition of LPS (Fig 5B). Under these conditions, the curcumin/liposome injection 4 h before harvesting the peritoneal cells evidently reduced IL-6 production. However, its administration 24 h before was less effective in the inhibition of IL-6 production. These results demonstrate that curcumin/liposome can at least suppress the IL-6-producing function in macrophages by in vivo administration in mice under proper conditions.

### Safety of curcumin/liposome for using in vivo in mice

**Fig 6:**
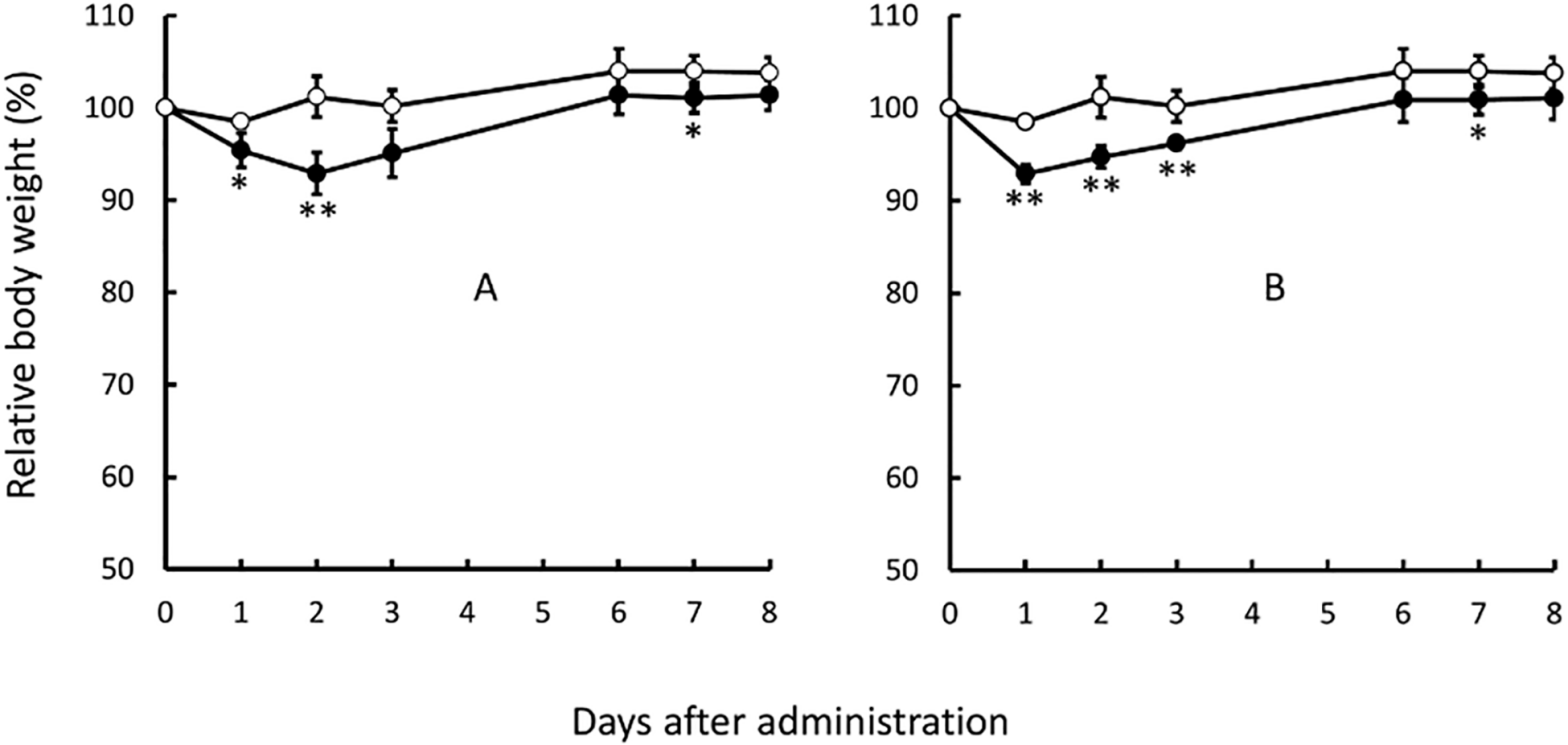
Effect of Curcumin/Liposome Administration on Body Weight Changes in Mice. Balb/c mice were injected i.p. (panel A) or i.v. (panel B) with curcumin/liposome suspension (200 μl). ○: untreated mice; ●: mice injected with curcumin/liposome (2.9 mg/ml). Data are shown as mean values ± standard deviation (bars) in five mice. The statistical analysis was carried out by the standard Student’s t-test. * and **indicate that P<0.05 and P<0.01 compared to untreated mice, respectively.

We examined the effect of curcumin/liposome administration on body weight and survival rates in mice. As shown in Fig 6, both i.p. and i.v. administration caused slight decreases in body weights for several days after the administration when compared to untreated mice. However, curcumin/liposome administration did not influence survival rates. Our results demonstrate that curcumin/liposome is much safer to use for suppressing macrophages in animals in vivo than clodronate/liposome, a known macrophage-suppressing reagent which causes death in particular after i.p. administration (data not shown).

## Discussion

In this study, we developed a novel method to suppress macrophages by utilizing a food ingredient and liposome. By in vitro culture assays, we chose curcumin for experimentation to develop a method for the suppression of macrophages. Curcumin reportedly has suppressive activities against inflammation in which macrophages participate [10, 29]. However, curcumin did not appear to have selectivity for macrophages in the suppression of cell proliferation. We thought DDS is essential for curcumin to use for the purpose of selective suppression of macrophages in vivo. In order to deliver curcumin to RES efficiently, we tried to prepare liposomes with curcumin. There are some reports on liposomes containing curcumin [11–13]. However, the properties of liposomal curcumin have not been delineated in detail. In this report, we examined various methods for preparing liposomes containing curcumin, and showed data on the difference of liposomes prepared by those methods (S1 Table). We succeeded in the preparation of liposomes containing a relatively high amount of curcumin (curcumin/liposome) by applying curcumin and albumin complex. Lipid composition of liposomes containing DPPC, DPPS, and cholesterol was chosen because this has reportedly been suitable for delivering to macrophages [30].

We analyzed the properties of curcumin/liposome. Its diameter distributed as a main peak of 60 to 100 nm. It has been proven that liposomes with this range of size could be efficiently delivered to RES in vivo in animals [4, 5, 9]. Z potential of curcumin/liposome was -30 mV, suggesting that it was negatively charged. We think this property (i.e., negative charge) is important to avoid nonspecific interaction of liposomal particles with cells in the body because particles with a positive charge will nonspecifically bind to cells with negative charge. It has been reported that curcumin destabilizes the structure of the cell membrane [31]. It remains to be clarified the exact distribution of curcumin/BSA in the nanoparticles.

To examine whether macrophages could selectively uptake curcumin/liposome in vivo, we administered i.p. into mice and analyzed peritoneal cells incorporating curcumin/liposome by flow cytometry. Because the majority of peritoneal cells are macrophages [27], we thought these cells are suitable to investigate the relationship between curcumin/liposome and macrophages. Under our experimental conditions, evident amplified fluorescence dots composed of curcumin and anti-macrophage marker (i.e., F4/80, CD36, or CD11b) antibody fluorescence were detected. These results demonstrated that mainly F4/80, CD36, and CD11b-positve macrophage incorporated curcumin/liposome. We believe that this is the first report demonstrating main peritoneal cells incorporating curcumin/liposome were macrophages.

Macrophages are one of the main producers of IL-6 efficiently in the body [28]. In fact, under our experimental conditions, peritoneal cells could more efficiently produce IL-6 than IL-1 or TNF (data not shown). As we expected, the addition of curcumin/liposome in the culture of mouse peritoneal cells drastically reduced LPS-induced IL-6 production at the range of 25 to 100 μM curcumin concentrations in in vitro culture. These results indicate that curcumin/liposome could suppress at least a part of macrophage functions. We subsequently tested the effect of curcumin/liposome in vivo in mice. After i.p. administering curcumin/liposome, we harvested peritoneal cells, and then examined the IL-6-producing ability of these cells in culture. Our results demonstrate that in vivo administration of curcumin/liposome evidently reduces the IL-6 production from peritoneal cells stimulated with LPS. Administration in vivo 4 h before the harvest of the peritoneal cells was more effective than that 24 under these experimental conditions. Besides, we observed the number of cells obtained from the peritoneal cavities in mice injected with crucumin/liposome before 4 h decreased to about half when compared with control mice (data not shown). These results suggest that curcumin/liposome could induce not only the suppression of IL-6 production but also the reduction of the number of macrophages, although we could not rule out a possibility that curcumin/liposome just changed cell trafficking in the peritoneal cavity.

When compared to clodronate/liposome, curcumin/liposome did not show drastic toxicity in mice both i.p. and i.v. administration. We examined the effect of curcumin/liposome administration on body weight and survival rates in mice. Both i.p. and i.v. administration caused very slight decreases in body weights for several days after the administration when compared to untreated mice (data not shown). However, curcumin/liposome administration did not influence survival rates. Our results demonstrate that curcumin/liposome is much safer to use for suppressing macrophages in animals in vivo than clodronate/liposome that causes death in mice by i.p. administration. We thus expect that curcumin/liposome is useful as a safer reagent for the selective suppression of macrophages in vivo in animals.

## Supporting Information

S1 Table Characteristics of Curcumin-Containing Liposomes Prepared by Various Methods. ^a^ Curcumin solution dissolved in 50% DMSO in water (v/v) was added to freeze-dried empty liposomes, and free curcumin and DMSO were removed by ultrafiltration [15, 16]. ^b^Curcumin was dissolved in 50% N,N-dimethylformamide (DMF; Wako) in water (v/v). Following procedure is described in the footnote^a^ [15, 16]. ^c^Curcumin was dissolved in 50% DMSO in water (v/v) containing 12% cremophore (w/v). Following procedure is described in the footnote^a^ [15, 16]. ^d^Curcumin was dissolved in water containing 22% cremophore (w/v). Following procedure is described in the footnote^a^ [15, 16]. ^e^ Transmembrane pH-gradient liposomes and curcumin were mixed at above the phase transition temperature (70°C) [17, 18]. ^f^Lipid films were hydrated with curcumin or curcumin/albumin solution [19]. ^g^ Curcumin or curcumin/albumin solution was admixed to a lipid suspension containing sodium cholate, and this surfactant was removed by ultrafiltration [20, 21]. ^h^ Initial amount of curcumin applied for liposomal procedure. ^i^Curcumin or lipid concentration after liposomal formation. ^j^These values were obtained by final curcumin concentration (μg/ml) / final lipid concentration (mg/ml). This is a parameter to evaluate loading efficiency of curcumin into liposomes. ^k^In freeze-drying or remote loading method, this represents each intraliposomal buffer of empty liposomes used for loading curcumin. In Bangham or improved cholate dialysis method, this represents each buffer used for hydration of lipid films. ^l^ Final suspension buffers of liposomes after loading curcumin.
(XLSX)
